# SCOLIONET: An Automated Scoliosis Cobb Angle Quantification Using Enhanced X-ray Images and Deep Learning Models

**DOI:** 10.3390/jimaging9120265

**Published:** 2023-11-30

**Authors:** Renato R. Maaliw

**Affiliations:** College of Engineering, Southern Luzon State University, Lucban 4328, Quezon, Philippines; rmaaliw@slsu.edu.ph

**Keywords:** atrous spatial pyramid pooling, computer vision, image enhancement, image processing, machine learning, medical image analysis, segmentation, spatial Wiener filter

## Abstract

The advancement of medical prognoses hinges on the delivery of timely and reliable assessments. Conventional methods of assessments and diagnosis, often reliant on human expertise, lead to inconsistencies due to professionals’ subjectivity, knowledge, and experience. To address these problems head-on, we harnessed artificial intelligence’s power to introduce a transformative solution. We leveraged convolutional neural networks to engineer our SCOLIONET architecture, which can accurately identify Cobb angle measurements. Empirical testing on our pipeline demonstrated a mean segmentation accuracy of 97.50% (Sorensen–Dice coefficient) and 96.30% (Intersection over Union), indicating the model’s proficiency in outlining vertebrae. The level of quantification accuracy was attributed to the state-of-the-art design of the atrous spatial pyramid pooling to better segment images. We also compared physician’s manual evaluations against our machine driven measurements to validate our approach’s practicality and reliability further. The results were remarkable, with a *p*-value (*t*-test) of 0.1713 and an average acceptable deviation of 2.86 degrees, suggesting insignificant difference between the two methods. Our work holds the premise of enabling medical practitioners to expedite scoliosis examination swiftly and consistently in improving and advancing the quality of patient care.

## 1. Introduction

**Figure 1:**
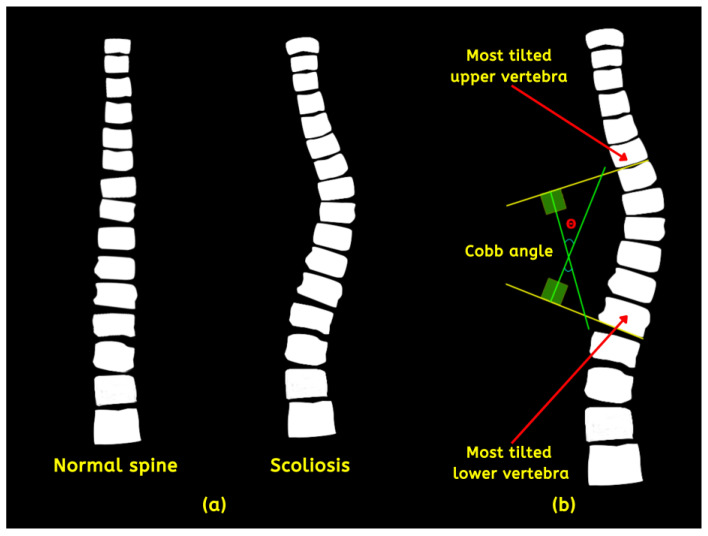
(**a**) Normal and abnormal spine and (**b**) detailed description of the method to acquire the CA.

The spine is the central pillar of the body’s structure that provides support, stability, and facilitates communication between various bodily systems. It comprises thirty-three (33) individual vertebrae, divided into five areas such as the coccyx (CO), sacrum (SA), lumbar (LU), thoracic (TH), and cervical (CE) [1]. Scoliosis is a medical condition distinguished by an irregular spine curvature, either inborn or developed throughout life due to other underlying factors [2]. The condition, if left untreated, can profoundly impact body posture, causing discomfort, pain, and in severe cases—paralysis. Moreover, it can affect cardiopulmonary function, compressing the lungs and ribcage, leading to breathing difficulties. Statistical figures report its prevalence from 510 to 5350 per 100,000 cases globally, most commonly during adolescence [3]. Although the abnormality’s origin is unknown and presents as mild conditions, some experience extreme situations, such as organ damage. The spine’s alignment should be straight (normal) and positioned centrally over the pelvis. On the other hand, scoliosis deviates from this norm, with a lateral curvature (left or right) that measures significantly more than 10 degrees [4]. Figure 1a compares the two instances. Doctors measure each case’s degree angles based on magnetic resonance imaging (MRI), computerized tomography (CT) scans, and X-rays to classify its severity using the Cobb angle (CA). Proposed predominantly by orthopedic surgeon John Robert Cobb and adopted by the Scoliosis Research Society (SRS), the CA is derived from selecting the two most tilted vertebrae depicted in Figure 1b.

The malformation is classified as mild, moderate, or severe. Table 1 shows the categorization per severity in terms of degrees.

**Figure 2:**
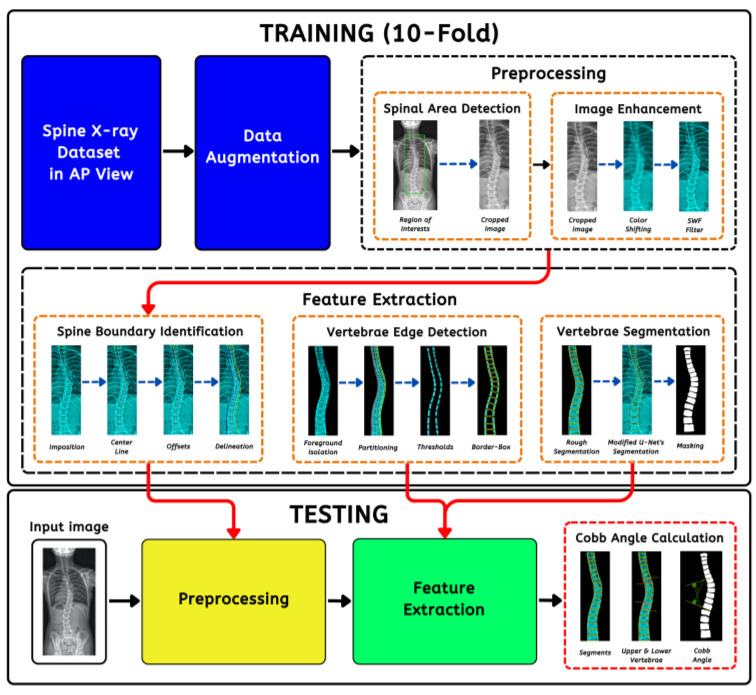
The automated scoliosis Cobb angle (CA) measurement’s comprehensive pipeline comprising preprocessing, feature extraction, testing, and angle estimation.

A conventional method of analyzing images to diagnose scoliosis involves extracting specific features from the scan, which is tedious and labor intensive. Accurately determining the extent of curvature through mechanical measurements can be challenging for physicians due to individual anatomical differences. In addition, traditional methods are subject to human error, leading to result inconsistencies [5]. Low-quality images and variations in patient’s posture and positioning during scans influence scoliosis assessments. Previous articles revealed three- to nine-degree deviations among medical professionals using manual methods [6]. Currently, artificial intelligence (AI) revolutionizes how we perceive and harness information [7,8,9] to improve image processing through its power to analyze vast amounts of data. It can assist in deciphering patterns for early and efficient diagnosis that proved difficult for humans to identify, paving the way for healthcare opportunities and discoveries. Despite various strategies for vertebral clustering and scoliosis measurement, the research domains are still in the early stages of development based on the literature. Most solutions are manual [10], patched-based, have parameter limitations [11,12], and do not consider each vertebra [13], thus losing crucial contexts. Huang et al. (2020) [14] published an article that utilized patch-wise portioning using minimum bounding boxes, allowing precise isolation. The study by Pasha et al. (2018) [15] utilized K-Means clustering for curvature modeling and regression for a vertebra’s corner detection; both required numerous preprocessing steps. Moura et al. (2016) [16] proposed techniques to recognize vertebrae’s lateral boundaries, including spine isolation. They removed other bone structures via progressive thresholding using tree data. Okashi et al. (2017) [11] used mice X-ray images to automatically subdivide and estimate curvatures with a three-stage process involving the Otsu algorithm, grayscale morphology, and polynomial fitting to refine spinal edges. Although innovative, the main disadvantage lies in its execution complexity without precisely measuring the CA. Mukherjee et al. (2014) [17] evaluated four denoising (bilateral, nonlocal means, principal neighborhood, and block) filters to enhance radiograph contrasts. Otsu thresholds and Hough transformation were employed for Canny edge points and vertebra endplate line overlays. Another experiment [18] incorporated scale-invariant features and support vector machines (SVM) for vertebral anterior corner tracking. The approach was promising. However, it was computationally intensive due to the intricate operations causing sizable errors. For the last few years, convolutional neural networks (CNNs) have been at the forefront of medical image processing (MIP) [19,20,21]. Unlike traditional machine learning (ML), they do not rely on handcrafted features for training. In detail, this means that neural networks (NNs) can intuitively extract and learn complex patterns with different levels of abstraction directly from the input data rather than requiring human experts to design attributes manually [22,23,24]. Additionally, a NN is end-to-end trainable. With better performance, they can autonomously optimize their layers for the task at hand, whether for object detection, semantic segmentation (SS), or classification [25,26,27]. Modern biomedical SS science has advanced significantly through the U-Net architecture [28]. In a nutshell, it uses unique encoder–decoder modules with a central bottleneck to capture local and global features ideal for image fragmentation. Arif et al. (2017) [29] applied different U-Net configurations to cluster cervical vertebrae with a Dice similarity coefficient (DSC) of 0.944 (shape aware) and 0.943 (standard). The outcome was a clear benchmark improvement against active shape model (ASM) segmentation types with ASM-G (0.774), ASM-RF (0.883), and ASM-M (0.877). A similar study was conducted by [30] using anteroposterior (AP) X-ray images. Results showed that the Residual U-Net (RU-Net) yielded better accuracy than the Dense U-Net (DU-Net), obtaining 0.951 DSC using RU-Net pitted against DU-Net’s 0.942. The use of non-standard deep learning models for image processing is crucial as it enables the tailoring of models to the unique requirements of specific domains, ensuring more accurate and practical solutions in various applications. It allows the customization of architectures to capture feature-specific patterns to address the many challenges more effectively than generic architectures. In resource-constrained environments, non-standard models can be designed to optimize resources, making them more suitable for deployment with computational efficiency [31]. As a contribution to data science advancement, we proposed a non-standard pipeline (Figure 2) codenamed SCOLIONET, composed of extensive preprocessing (cropping, color adjustments, and image enhancements) and a robust modified segmentation architecture with a new atrous spatial pyramid pooling (ASPP) structure to quantify CA accurately. Our initiative can contribute to swift, consistent, and prompt scoliosis severity diagnosis.

## 2. Methodology

This section presents comprehensive details of the procedures involved in automatically acquiring CA measurements.

### 2.1. Data Collection

Our dataset contained 318 two-dimensional (2D) spinal X-ray scans, specifically in the anterior–posterior (AP) view, showcasing scoliosis. In grayscale format, these photos had various resolutions. We meticulously curated the samples from various public repositories without traces of personal information, in compliance with the state’s data privacy decrees (see data availability statement). The samples encompass visuals of lumbar and thoracic parts; a prerequisite for the execution of the designed processing procedures with ten validations, 10% test sets (20), and 90% training sets (288). Every dataset had its corresponding observed CA measurements annotated by experts for benchmarking of our deep learning (DL) approach against conventional methods.

### 2.2. Spinal Region Isolation

**Figure 3:**
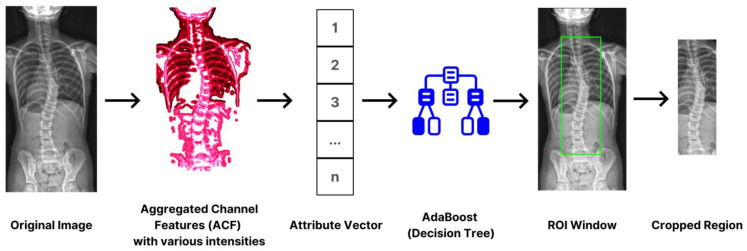
The process of spine region isolation using ACF and AdBoost to reduce the inputs’ dimension.

Determining the focus area or the ROI (region of interest) is crucial in reaching our intended outcome. This essential method significantly reduces and eliminates substantial noise, improving results. We trimmed the size to approximately thirty percent of its original dimension, focusing on the thoracic and lumbar vertebrae. These parts, based on statistics, were susceptible to scoliosis. To accomplish the tasks, we utilized an aggregated channel feature (ACF) in LUV mode, enabling the extraction of pixel-based features directly from color channels and gradient magnitudes. Utilizing the scheme offers a clear advantage. Primarily, we employed an adaptive boost, commonly known as AdBoost. By incorporating the classifier, we discerned the patterns associated with spinal images. As a final step, it concludes with a cropping operation based on the ROI, isolating the locality for further analysis. This procedure not only optimizes computational efficiency by narrowing down the spatial emphasis but also ensures that the segmentation process was conducted in relevant areas only. Figure 3 provides the flow of operation.

### 2.3. Color Standardization and Image Enhancement

**Figure 4:**
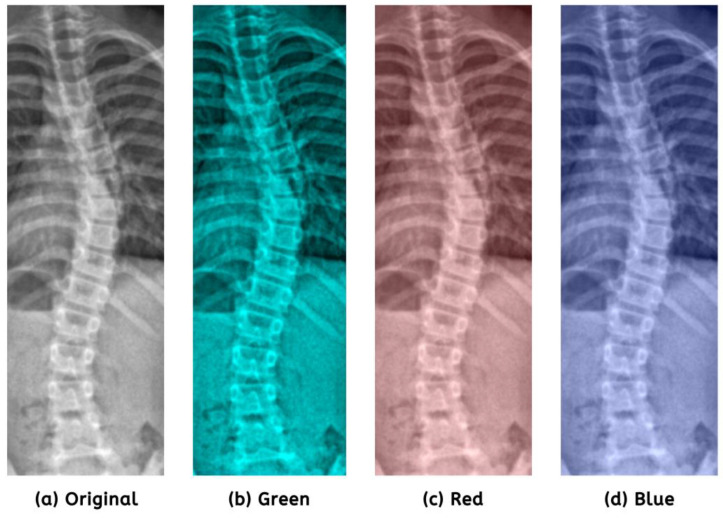
Prioritization of the green channel (**b**) had shown minor detail improvements.

Regarding image processing, one must recognize the significance of color shifting, as it improves visual quality. It increases interpretability by emphasizing essential features and suppressing noise, enabling algorithms to analyze and examine images more effectively. In preparation for image enhancement, we refined the ideal color settings using specific values: red = 0.21, green = 0.59, and blue = 0.20. Figure 4 illustrates the comparison of color channels.

Distinguishing anatomical differences in chest X-rays can be challenging due to the overlapping structures and intersecting details (e.g., bones and organs). We performed image enhancement procedures to address this issue to increase contrasts caused by noise and blurring using a spatial Weiner filter (SWF). Equation (1) express an original image (OI) *a*(*x*, *y*), containing noise or blurring *n*(*x*, *y*), and a noisy image (NI) *z*(*x*, *y*) [32].

(1) z(x, y)=a(x,y)+n(x, y)

The noise, which is assumed to be stationary, is described by a zero mean and variance $\delta_{n}^{2}$. Also, the noise is independent of the OI described by Equation (2) [32]:

(2) $$o\left(x,\;y\right)=m_{s}+\delta_{s}w\left(x,\;y\right)$$

Localized entities $m_{s}$ and $\delta_{s}$ represent the mean and standard deviation in proximity, with w(x, y) denoting zero-mean noise variance. The SWF efficiently minimizes the mean squared error between the OI and the enhanced image $\overset{´}{z}\left(x,y\right)$ calculated from Equation (3) [32]:

(3) $$\overset{´}{z}\left(x,\;y\right)=m_{s}+\frac{\delta_{s}^{2}}{\delta_{s}^{2}+\delta_{r}^{2}}\;\left[a\left(x,y\right)-m_{s}\right]$$

At each pixel, $m_{s}$ and $\delta_{s}$ are updated using Equations (4)–(6), which estimate their values based on NI [32].

(4) $${\hat{m}}_{s}\left(x,\;y\right)=\frac{1}{\left(2e+1\right)\left(2f+1\right)}\;\overset{i+e}{\underset{k=i-e}{\sum }}\overset{a+f}{\underset{l=a-f}{\sum }}v\left(k,\;l\right)$$

(5) $${\hat{\delta }}_{a}^{2}\left(x,\;y\right)=\frac{1}{\left(2e+1\right)\left(2f+1\right)}\;\overset{i+e}{\underset{k=i-e}{\sum }}\overset{a+f}{\underset{l=a-f}{\sum }}{\left[v\left(k,\;l\right)-{\hat{m}}_{s}\left(x,\;y\right)\right]}^{2}$$

(6) $${\hat{\delta }}_{s}\left(x,\;y\right)=max\left\{0,\delta_{a}^{2}\right\}\left(x,\;y\right)-\delta_{r}^{2}\}$$

Afterward, Equation (3) integrates the substitutions of ${\hat{m}}_{s}\left(x,\;y\right)$ and ${\hat{\;\delta }}_{s}\left(x,\;y\right)$ as part of each iteration, leading to:

(7) $$\hat{o}\left(x,\;y\right)={\hat{m}}_{s}\left(x,\;y\right)+\frac{{\hat{\delta }}_{s}^{2}\left(x,\;y\right)}{{\hat{\delta }}_{s}^{2}\left(x,y\right)+\delta_{r}^{2}\;}\;\left[z\left(x,\;y\right)-{\hat{m}}_{s}\left(x,\;y\right)\right]$$

**Figure 5:**
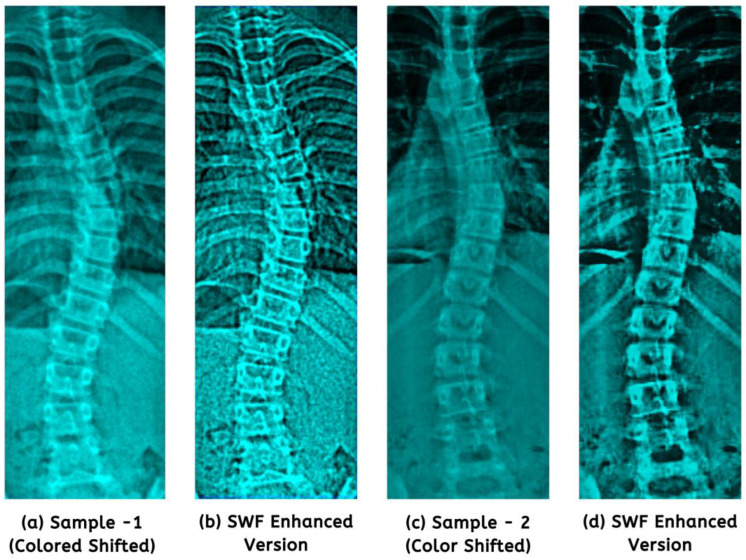
The application of the SWF revealed structural and finer enhancements (**b**,**d**) from colored shifted images.

In the end, we established a fixed filter size of 3 × 3 (from 2e + 1 and 2f + 1) based on experimentation. Noteworthy improvements in structural spinal details are displayed in Figure 5. It presents a visual testament to the enhanced images used for model training.

### 2.4. Spinal Boundary Detection

**Figure 6:**
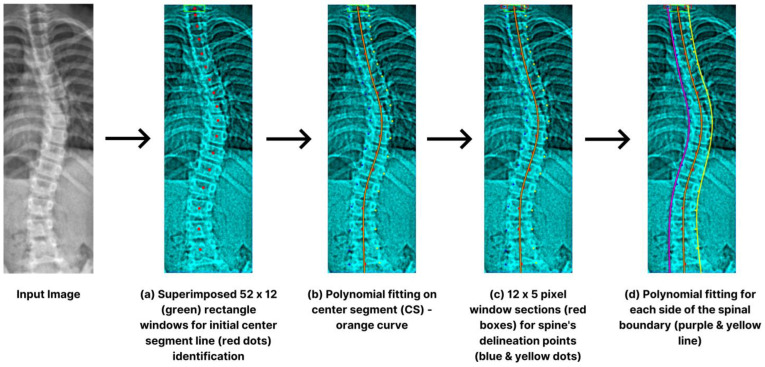
Step-by-step procedures for spinal limit detection such as superimposition (**a**), midline polynomial fitting (**b**), delineations (**c**), and boundary polynomial fit (**d**).

We used the ROI image for accurate vertebra localization upon successful spinal region isolation. Intensity values capture the brightness of each pixel. We extract specific color information to enhance edge detection, where distinct color features characterize different anatomical structures. The initial stage involves crafting a specific vertebral section serving as the center line (*CS*). Rectangular windows, defined by height (*H*) and width (*W*), were overlaid horizontally in one-pixel increments from the apex of the spine [33]. The computation involved determining the luminosity within each rectangle to establish the *CS* reference point shown in Figure 6a. Subsequently, we initiate a downward displacement of the rectangular frame that exhibits the highest intensity, changing its position by a pixel. We aimed to pinpoint explicit pixels on each side, using distance (*q*). By conducting iterative operations, we were able to identify multiple reference points (*r*). This *r* was then fitted into *CS* via polynomial fitting, as illustrated in Figure 6b.

For the exploration of the spine’s delineation points in a downward direction, we utilized small widow sections (12 × 5 px), traversing (*x*) the identified *CS* depicted in Figure 6c. To ascertain the spine’s boundaries, we selected the midpoints with the most substantial intensity difference between window frames. The process iteratively examined all potential touch points (*r*) along the *CS*. The *CS*’s endpoint matching window was reconstructed, enabling sequential spinal limit detection. A 4-degree polynomial fit on each side assists the process (Figure 6d). For the identification of the edges, we experimentally set the following hyperparameters: *W* = 12, *H* = 52, *x* = 36, *r* = 5, *q* = 12, and *p* = 11.

### 2.5. Initial Vertebra Identification

**Figure 7:**
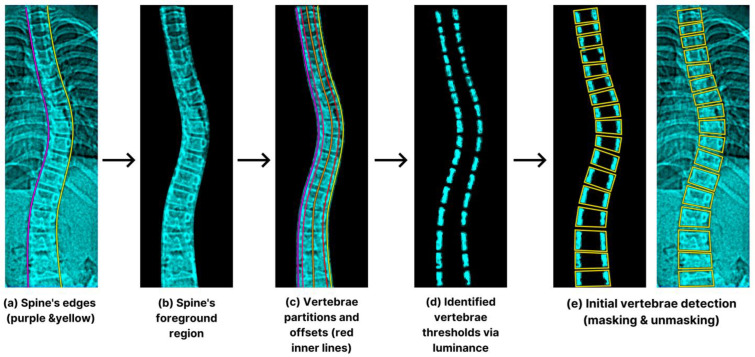
The process starts with spine edge determination (**a**), foreground isolation (**b**), partition and offsets (**c**), luminance thresholds (**d**), and vertebra detection (**e**).

Once the spine’s edges’ definition was in place (Figure 7a), we isolated the foreground region displayed in Figure 7b to eliminate unwanted anatomical structures. This critical process facilitates the preliminary vertebra selection by using four equal sub-spaced lines showcased in Figure 7c,d to generate sets of values and thresholds. In addition, we noticed greater luminosities within the vertebrae’s outer borders and mathematically represented their histogram projection (*p_t_*) using Equation (8) [33]:

(8) $$f_{t}\left(h\right)=\int_{1,\;opposite,}^{0,\;if\;p_{t}\left(h\right)>0}$$

where *h* is the histogram value with a constant *B* for the histogram’s (*p_t_*) bin dimension; the summed histogram (*S*) is the subtotal of each feature *f_t_* illustrated in Equation (9) [33]:

(9) $$S\left(h\right)=\overset{n}{\underset{i=1}{\sum }}f_{t}\left(h\right)$$

The facet of *S*’s calculation lies in the active involvement of adjacent disc pixels, with the predominant presence of zero (0) values. By selecting significant ascending shifts in *S*, the algorithm identified various reference points. Next, we configured an 18-bin sub-histograms (non-overlapping) beginning from the lower boundary. The final vertebral ROI was enclosed by the contiguous straight lines shown in Figure 7e.

### 2.6. SCOLIONET’s Detailed Core Network Architecture

**Figure 8:**
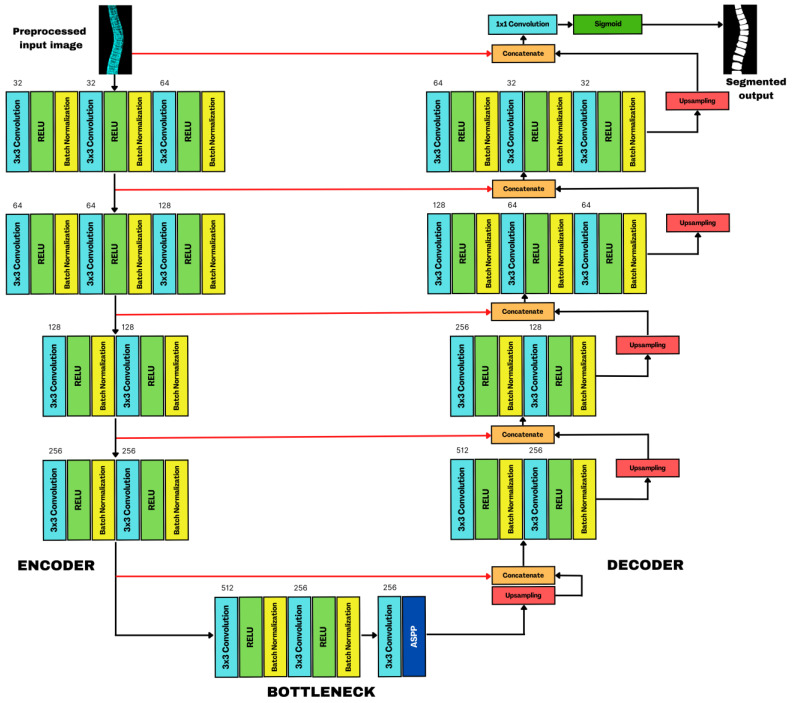
SCOLIONET’s architecture composed of encoder, bottleneck with atrous spatial pyramid pooling (ASPP), and decoder for spine’s vertebra segmentation.

With the initial individual vertebra determination completed, a finer ROI was extracted. It is worth highlighting that each vertebra’s intensity varies considerably in its AP projection. The lumbar portion exhibits higher intensity, whereas the cervical part displays lower. CNNs are robust to different lighting conditions as they can effectively extract features from images, making them less sensitive to saturation than other techniques. We customized and tweaked a Standard U-Net as a solution because it was designed for general segmentation tasks and was unsuitable for our purpose after multiple tests. Our architecture is composed of three parts (Figure 8). On the left side, a four-block (BLK) encoder takes the input image while the next BLK encoder takes the input image. The next BLK receives the previous output subsampled at a lower rate. The first two convolution (CNV) layers have thirty-two (32) feature maps (FM), while the third contains sixty-four (64) FM. Like its predecessor, the second unit follows a similar configuration. It features sixty-four (64) and one-hundred-twenty-eight (128) FM across its layers. The third and fourth BLK seamlessly integrate two CNVs, doubling subsampled FM, reaching 128 and 256. At the end of each BLK, maxpooling (MP) downsampled the spatial dimension of FM, this helps reduce computational complexity and memory usage. It retains the essential information from the original FM while helping the network to focus on the most discriminative features and to discard spatial redundancies. At the onset of each MP, the network creates a copy as a skip connection to ensure that high-resolution FM from the contracting path is passed directly to the corresponding layer. Doing so retains the final spatial information lost during downsampling, leading to segmentation precision. The decoder on the right follows the same architecture as the encoder, concluding with a 2D upsampling technique as it needs to restore the information produced by the segmentation mask. In Figure 8, the red arrow is indispensable for reconstructing stored FM in the encoder block’s cluster layer. These serve as the basis for comparison with the decoder’s oversampled outputs, increasing its ability to segment in great detail each vertebra through concatenation. A sigmoid activation function and a 1 × 1 kernel convolution complete the process and the FM’s outcome.

The bottleneck in the middle is a bridge between the two pathways with fewer channels than the encoder and decoder. It contains a MP, a 3 × 3 kernel applied to the input to extract features, and a 2 × 2 kernel with stride extrapolated to 2 × 2 regions with a pooling window with two pixels at each step, thus enriching the FM and helping the network to learn intricate and abstract features. A rectified learning unit (ReLU) activation function helps introduce non-linearity for learning of complex relationships in data while mitigating vanishing gradient problems. Batch normalization (BN) helps prevent extensive activation and decrease covariate shifts to provide stability during the training. As a prime customization, we integrated atrous spatial pyramid pooling (ASPP) to improve training speed, increase the receptive field, capture fine details, and strengthen the architecture’s segmentation efficiency without added parameter overheads. Performance of our SCOLIONET, U-Net, and RU-Net were rigorously compared. To assess the quantitative efficiency of segmentation, we performed a 10-fold cross-validation. For the hyperparameters, the following were set based on trial and error: batch size = 12, epoch = 120, learning rate = 0.01, dropout rate = 0.20, and an L2-norm loss function to improve segmentation further.

### 2.7. Atrous Spatial Pyramid Pooling (ASPP) Structure

**Figure 9:**
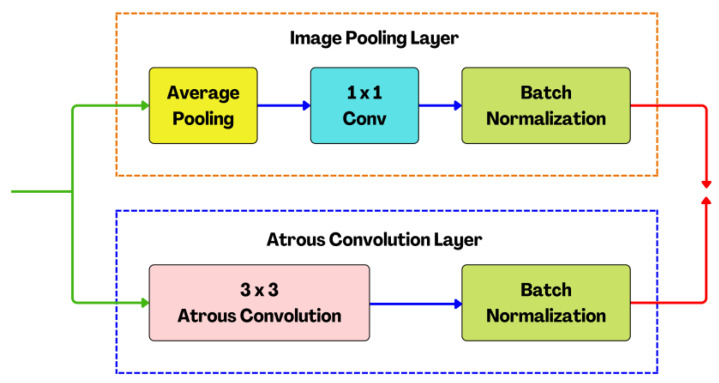
The structure of the proposed atrous modules (AM) with the atrous convolution layer and image pooling layer.

Feature pyramid network (FPN) core strength lies in its ability to seamlessly integrate semantic insights from low-resolution feature maps with the intricate spatial details extracted from high-resolution feature maps. The combination of semantic information from lower-resolution levels and spatial intricacies from higher-resolution levels creates a holistic representation of the visual content. This synergy is significant in terms of object detection and segmentation, where objects of interest can vary significantly in size within an image. By fusing these distinct types of information, FPN equips neural networks with a clear understanding of both the global context and fine-grained details present in the visual data. It mimics the human visual, where our brain effortlessly integrates coarse and detailed information to form a complete perception. FPN, in its computational parallel, enables machines to achieve a similar level of perceptual completeness. Furthermore, FPN enhances the efficiency of information flow through the network, optimizing computational resources and facilitating more accurate and context-aware predictions. This not only improves the accuracy of tasks such as object recognition, but also contributes to the model’s ability to generalize well across diverse datasets. In this study, we proposed an FPN by incorporating atrous modules (AM) consisting of atrous convolution and an image pooling layer (Figure 9). The design philosophy behind these modules is akin to creating a dynamic, multi-scale receptive field by allowing the neural network to perceive and analyze features at varying granularities. Through this integration, the resultant feature representation becomes adaptive to discern complex structures within the input data. The module is an intentional departure from conventional, uniform convolutions, showcasing our commitment to a robust feature extraction.

**Figure 10:**
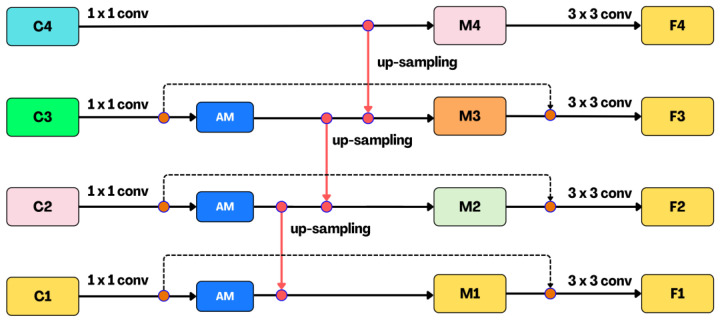
The proposed architecture of a feature pyramid network with atrous modules (AM) with additional edges, F1 to F4 are the fused output of M1 to M4 with the same dimensions.

The primary objective is to leverage both spatial and semantic information more effectively within the network architecture to enhance the model’s ability to comprehend and interpret intricate patterns and structures within the data. We aim to strike a balance between capturing fined-grained spatial details and grasping the broader context of semantic meaning. This is pivotal, especially in a task where understanding both the details and the overarching semantics of the input data is essential. The implemented adjustments ensure that spatial nuances are preserved and integrated cohesively, with semantic understanding at various levels of the feature hierarchy. Integrating the AMs into the FPN, X1 to X4 undergoes a 1 × 1 convolution for optimizing them for the subsequent fusion process. Next, C1 to C4 go through the AMs with atrous rates at 2, 4, and 6. When dealing with relatively small scales, the use of rates becomes instrumental because a 3 × 3 filter convolution can lose its efficiency and degenerate into a 1 × 1 filter if the rates become larger. Understanding and optimizing these atrous rates are essential for preserving the hierarchical information with feature maps. The integration of high-level and low-level features, specifically from F1 and F4, is achieve through additional edges, which are then subjected to a summation process facilitated by a 1 × 1 convolution layer. Importantly, this fusion is executed to increase the model’s capacity without inflating computational complexity. By using a 1 × 1 convolution layer, the fusion of features is performed with a minimal increase in the number of parameters, contributing to a streamlined and effective neural network architecture. Finally, C1 to C4 undergo a 3 × 3 convolution independently. The purpose of this operation is twofold: First, it serves to remove the aliasing effect during upsampling, ensuring fidelity of features. Second, by conducting these convolutions independently, we preserve the unique characteristics of each channel. Its design not only mitigates potential instability but also aligns with the principle of preserving essential information as it traverses through the network. Figure 10 shows our pyramid network.

### 2.8. Cobb Angle Reference and Calculation

**Figure 11:**
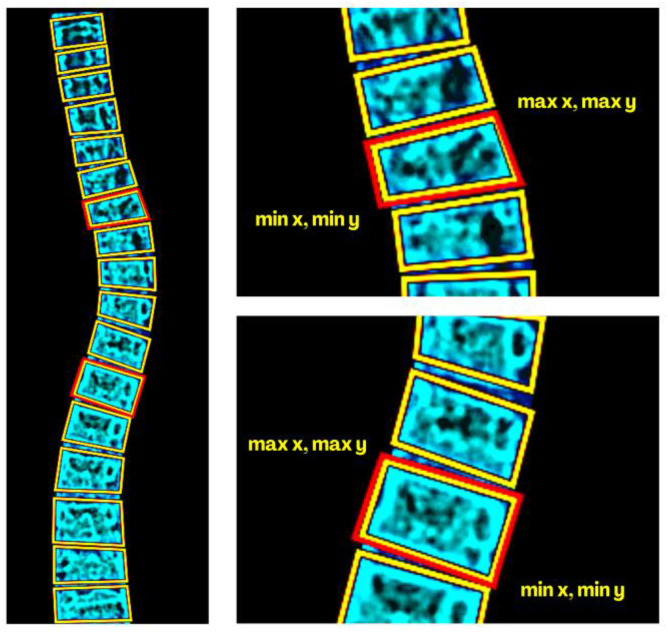
Border-box methods (BBM) for determining the most tilted endplates of the referenced upper and lower vertebra’s border.

After a fined-tuned segmentation, the contours were extracted to represent boundaries through a bounding box method (BBM). These boxes were then stored in an array (maximum x, minimum y, maximum y, and minimum y) illustrated in Figure 11. Using the array’s values, the method identifies the lower and upper borders of the vertebrae. Then, the angles of the endplates (flat surfaces at the top and bottom of each vertebra) were re-stored in another array. These angles were significant in identifying tilted vertebrae as they convey reference points for the analysis. The process was iterative by comparing the adjacent endplate angle differences, ensuring the determination of the largest angle. CA is then calculated using Equation (10) [33]:

(10) $$CA=\max\left\{\left|tan^{-1}\left(\frac{z_{i}-z_{j}}{1+z_{i}-z_{j}}\right)\right|\right\}$$

where *Z_i_* and *Z_j_* are the slopes of the upper and lower edges of the identified reference vertebrae, respectively.

### 2.9. Quantitative Image Enhancement Evaluation

Traditionally, the assessment of image quality improvement relied on manual visual inspection. However, this approach is subjective and inconsistent. To address this limitation, we used visual information fidelity (VIF) for an objective and concrete evaluation of graphics improvement. VIF is a specific metric designed to measure digital enhancement quality that is more rigorous and quantitative by accounting for various attributes [34]. Its process compares the original and processed images through overlapping blocks to form a single VIF score scaled from 0 to 1. A higher value indicates a better picture quality. This scaling ensures consistency and reliability, making it valuable where precision is essential. We utilized correlation coefficient (CC) and Spearman rank order *CC* (SROCC) considering numerous factors influencing the perceived visual fidelity. Radiologists suggest a VIF score of 0.9 to 1 as an excellent improvement. The general formula is expressed by Equation (11) [35]:

(11) $$\mathrm{VIF}=\frac{\sum_{j\in sub-band}I\left({\bar{C}}^{N,\;j};\;{\bar{F}}^{N,\;j}\;|\;S^{N,\;j}=s^{N,\;j}\right)}{\sum_{j\in sub-band}I\left({\bar{C}}^{N,\;j};\;{\bar{E}}^{N,\;j}\;|\;S^{N,\;j}=s^{N,\;j}\right)}$$

where *N* are small overlapping blocks as part of the sub-band decomposition, denoted by *j.* The ${\bar{C}}^{N,\;j}$ represents resemblance variables associated with specific j∈sub−band.

### 2.10. Segmentation Assessment Measures

We calculated the segmentation’s accuracy of results using intersection over union (IoU), the Sorensen–Dice coefficient (SDC), and mean squared error (MSE). IoU is a commonly applied metric to evaluate segmentation algorithms’ performances, specifically in computer vision (CV) tasks. It calculates the degree of overlap between ground truth (GT) and predicted regions (PR). For each image’s object, GT represents the actual location and PR generated by the segmentation algorithm. As expressed in Equation (12), it is calculated by dividing the intersection by the union’s area, where the value ranges between 0 (no overlap) and 1 (perfect match).

(12) $$IoU=\frac{\left|GT\cap^{\;}PR\right|}{\left|GT\cup^{\;}PR\right|}$$

Another mathematical framework evaluation is the SDC. At its core, the SDC measures the extent of the agreement by identifying the common elements between two objects by dividing the size of the overlap score by the sum of the dimensions of two segmented regions (Equation (13)). As an advantage, it is scale invariant, robust to class imbalances, threshold independent, and highly interpretable.

(13) $$SDC=\frac{2\left|GT\;\cap^{\;}PR\right|}{\left|GT\right|+\left|PR\right|}$$

Lastly, we used the MSE to quantify the squared differences between GT and PR at the pixel level (Equation (14)). The MSE regards minor and major differences, penalizing larger deviations and making the calculation sensitive to significant changes for spotting subtle variations. Furthermore, it can offer consistency on a large-scale evaluation.

(14) $$MSE=\frac{1}{n}\;\overset{n}{\underset{i=1}{\sum }}{\left(GT_{i}-PR_{i}\right)}^{2}$$

### 2.11. Degree of Difference Evaluation Metrics

In this article, we compared the medical expert’s annotated (manual) and our AI approach CA measurements. We conducted a *t*-test on normally distributed data, a well-established statistical technique to ascertain the validity of our findings. The objective is to identify substantial differences between two distinct and independent groups. We used a *p*-value (pv) threshold of 0.05 and confidence interval of 95%. A pv lower than the configured parameter strongly indicates notable divergence. At the same time, a higher value suggests significant indifference. The formula is represented by Equation (15):

(15) $$t=\frac{\bar{g_{1}}-\bar{g_{2}}}{\sqrt[{S_{p}}]{\frac{1}{n_{1}}+\frac{1}{n_{2}}}}$$

where $\bar{g_{1}}$ and $\bar{g_{2}}$ are the means of two groups, $n_{1}$ and $n_{2}$ are the group’s sample sizes, with $S_{p}$ as the pooled standard deviations.

### 2.12. Cobb Angle Measurement’s Reliability Test

We assessed our angle measurements with a clear intention of enhancing reliability. Our motivation for another metric was to ensure our calculations were consistent and dependable. To accomplish this, we used a mean absolute percentage error (MAPE), known for its relevance as a scale-independent (percentage-based) measure for most prediction scenarios. In addition, it is less sensitive to outliers, allowing a straightforward, clear, and understandable interpretation for general audiences. The formula is depicted by Equation (16):

(16) $$MAPE=\frac{\sum_{i=1}^{N}\;\left|\;\frac{a_{i}-x_{i}}{x_{i}}\;\right|\times 100}{N}$$

With $a_{i}$ and $x_{i}$ the actual and predicted values, and N as the total number of samples.

## 3. Results

We conducted our experiment on a computer with an AMD Ryzen 9 5900X processor (4.8 Ghz, 64M cache), 64 GB DDR4, and a GeForce RTX3080 graphics processing unit (1.71 GHz, 20 GB). It also boasts a 1 TB NVMe solid-state drive (SSD) and a 4 TB hard disk drive (HDD). Although there are two storage devices at our disposal, we opted to use the SSD for its near instantaneous access times, leading to quicker loading of training images and faster processing. Moreover, we used TensorFlow and other related libraries for the deep learning network’s construction. Subsequent sections present the detailed results.

### 3.1. Image Enhancement Performance Evaluation

To comprehensively evaluate our enhancement approach, we subjected 100 randomly selected images to quality assessments. As outlined in Table 2, notable improvements were observed in mean outcomes for raw input images processed with color shifting and SWF. The result showcased a highly similar CC value of 0.973 and a positively monotonic SROCC of 0.968. The preprocessed AP scans revealed more subtle details and distinguishable features while preserving the overall integrity of the images’ structures (Figure 5), a precursor for diagnostic reliability.

### 3.2. Computing Performance Assessment

The measurement of computational performance in neural networks has far-reaching implications in research and practical applications. By conducting this evaluation, we can ascertain the extent to which computational resources are utilized and how they can provide insights into the networks’ capability to operate within the constraints of the computing environment. In the context of scalability, the metrics serve as a critical indicator of a model’s adaptability to varying scales of data and computational infrastructure. Table 3 presents our SCOLIONET’s computational load based on processor cores. The data suggest that, in general, increasing the number of active processor cores from eight results in a reduced training time for all three models. This aligns with the parallel processing power of multi-core systems. In terms of training time, it varies between the architecture’s complexities. Residual U-Net and SCOLIONET, in this case, show longer training times compared to the Standard U-Net.

Memory constraints can significantly impact the efficiency of the training process. In scenarios where models need to scale across distributed systems or edge devices, efficient memory usage is important. We use the nvidia-smi command to retrieve vital information from the NVIDIA graphics processing unit (GPU). It is imperative to note that, during model inference, the memory consumption of the GPU can be accurately ascertained by examining the results from the application programing interface (API). This serves as a reliable indicator of the GPU’s memory usage throughout the inference process regarding resource allocation and overall system performance monitoring. Table 4 depicts the memory consumption of the selected models based on number of batches. The memory utilization for Standard U-Net remains relatively stable across different sizes, ranging from 0.63 to 0.68. It suggests that the model does not exhibit sensitivity to changes in batch size. On the other hand, the Residual U-Net, while starting with a slightly higher base line of 0.72, maintains consistency within a reasonable range with batch sizes of 0.66 to 0.78. However, there is a noticeable increase in memory usage as the batch grows, indicating that the model is resource demanding with larger batches. SCOLIONET’s consumption is fairly similar to the Residual U-Net at lower batches but demonstrates a distinct pattern. Its memory consumption is relatively low at a batch size of 1 (0.72) and gradually increases. The model appears efficient in handling individual instances but experiences a proportional rise in memory demands as the batch size expands.

### 3.3. Segmentation Performance and Visual Confirmation Evaluation

**Figure 12:**
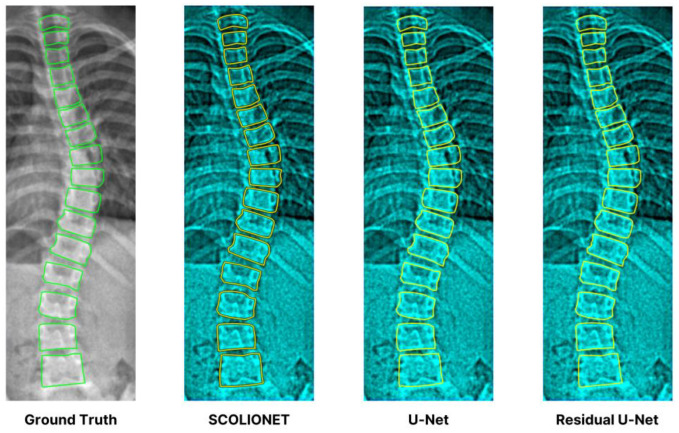
Visual segmentation excerpt results of the three models showing SCOLIONET’s capability.

Table 5 presents the evaluation results of SDC, IoU, and MSE obtained from the CNN segmentation models’ cross-validation. The results disclose that SCOLIONET (0.975, 0.963, and 0.025) performed better than a RU-Net (0.950, 0.942, and 0.030), and U-Net (0.941, 0.926, and 0.032) and in achieving overall vertebra segmentation accuracy. Moreover, Figure 12 complements and reinforces the quantitative measures by providing an excerpt of the visual representation produced by the three architectures with references against the GT.

### 3.4. Cobb Angle Performance Evaluation

**Figure 13:**
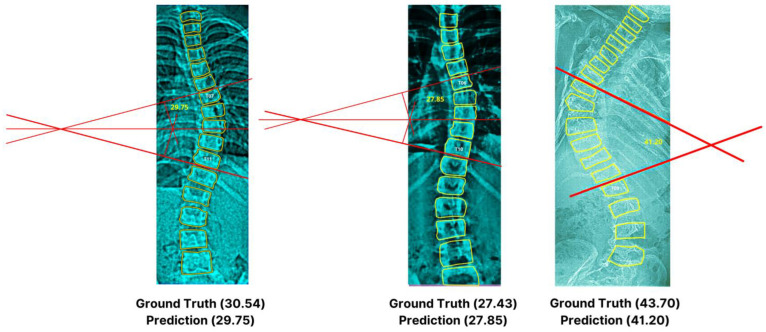
Excerpt of Cobb angle measurements with computer generated reference lines.

Table 6 demonstrates the detailed outcome of our deep learning approach versus manual measurements. Notably, the *t*-test emphasized no significant differences between the two groups, highlighted by a *p*-value = 0.8659 and a t-value (degree of freedom = 18) of 0.1713. This convergence is evident through the MAPE of 3.86% or 96.13% accuracy. The findings affirm our model’s close alignment with the actual observed annotated values, showcasing an impressive 2.86-degree discrepancy (very small). Although the angle deviations are small for minor scoliosis cases, it can be observed that, as the curvature increases, it can become challenging for the algorithm to precisely identify the end vertebrae that can be susceptible to errors [36], as can be seen on X-ray IDs 0203 and 0253 with a difference of a 2.20 degree angle from the actual values (inter- and intra-observer variability). Furthermore, an excerpt of the visual representation depicted in Figure 13 exhibits our artificial intelligence-based technique for various angles.

### 3.5. Benchmarked Performance versus Existing State-of-the-Art Approaches

Table 7 presents the performance of different architectures, models, and special mechanisms for vertebra segmentation.

Despite being a foundational architecture, the Standard U-Net achieves a reasonable accuracy (88.01%). The variation in configurations suggests the importance of hyperparameter tuning for optimal performance. A patched-wise approach demonstrates a slight improvement in accuracy (88.60%). Next, the K-Means clustering and regression offers efficacy of the unsupervised method (clustering) with the introduction of regression for refinement (88.20%). Residual U-Net (88.30%) and lateral boundary detection (88.50%) highlight the importance of detecting boundary information for precise segmentation. A multistage Otsu (90.20%) and Hough transformation showcases a refined technique where precise delineation is crucial (90.30%). With corner tracking combined with SVM, it demonstrates an even better machine learning for image segmentation (90.40%). With the advancement of algorithms such as Dense U-Net, having dense connectivity patterns substantially improves accuracy, underscoring the importance of information flow between densely connected blocks (94.20%). The polynomial fitting method and minimum border-box techniques with Residual U-Net leads to accuracy leap, demonstrating the synergistic effect of combined approaches (95.10%). Lastly, our SCOLIONET architecture with comprehensive preprocessing and non-standard techniques achieves the highest accuracy of 97.50% by incorporating different strategies for comprehensive segmentation.

## 4. Discussion

The findings of our research represent a noteworthy breakthrough, establishing the capability of our method to automatically quantify CA, pivotal information for determining scoliosis severity. Based on experimental results, our SCOLIONET beats U-Net and RU-Net with segmentation accuracies of 97.50% (SDC), 96.30% (IoU), and 0.025 (MSE). A reported reduction in segmentation accuracy by 1.92%, or almost two percent, translates to a marked improvement rate, especially considering the complexities and intricacies often encountered in image processing. Empirical metrics also show a 95.86% accuracy based on MAPE (3.86%). The insignificant difference between the AI-powered automated method (*t*-test *p*-value = 0.8659) and the traditional technique validated the entire pipeline’s robustness. Various factors collaboratively led to this outcome. First, the integration of color shifting and SWF for image enhancement amplified the visual information inherent in the raw X-ray without compromising intrinsic structural integrity. These added vibrancy and depth to the images serve as crucial markers for deep learning. Second, modularizing the procedures from spinal isolation, spinal edge detection, and vertebra segmentation diminished unnecessary overheads in the training and learning phase. Third, the customization and inclusion of ASPP in the U-Net’s architecture increases segmentation accuracy and its ability to capture multi-scale contextual information. It adds discriminative power to the network to identify subtle or minute pixel classification in most spinal images. Lastly, our examination unveiled that a network’s complexity (RU-Net) does not innately translate to superior segmentation performance. This realization underscores a fundamental principle in computer vision, that the model’s efficiency is tied to its alignment with the specific demands of the application at hand. By drawing parallels between our findings and existing research on machine learning-based medical image diagnosis, we have collectively made a conscious effort to build upon the knowledge amassed by our predecessors. We also acknowledged the ongoing evolution of the field and positioned our work within the continuum of advancements in automated medical image processing. Like most studies, we confronted various challenges internal to scoliosis assessment.

While our research focused on moving the field forward, it is essential to divulge that our efforts have limitations, mainly when dealing with images of highly deficient quality. It is also important to note that our collected data are limited to a fully developed spine with ages 16 and above, marking the end of significant longitudinal bone growth. In reality, human vision is a highly dynamic sensory perception that understands depth, colors, motion, complex patterns, and prior experiences. While computer vision is becoming sophisticated, often focusing on object detection, image segmentation, or facial recognition. These systems still lack the holistic and contextual understanding that human vision possesses such as creativity that cannot be captured by predefined rules and patterns created by algorithms. When human expertise is combined with machine knowledge to aid in diagnosis, the challenges of ambiguity can be blurred. Additionally, patient’s unbalanced positions and postures during image scans could introduce deviations in the CA. We recognized that these uncertainties were common to image processing and not directly addressed by our current approach. These avenues present valuable opportunities for further research and innovation to achieve a more accurate measurement in a broader range of clinical scenarios.

## 5. Conclusions

Scoliosis, a spinal abnormality, poses an expanse of health-related adversities, from short- to long-term complications. It includes posture deformities, balance issues, degenerative diseases, and potential harm to internal organs. For this context, accurately gauging the severity of spinal curvature is paramount for medical practitioners, serving as valuable informational support for effective treatment planning. Historically, measuring the CA—a key indicator of scoliosis—has been a painstaking mechanical process. It is prone to disparities linked to subjective factors of a physician’s training, experience, and case-specific expertise. The complications of manual vertebra segmentation and referencing, often reliant solely on the human’s naked eye, introduce further difficulties. Moreover, the implicit noise with X-ray images with the intersection of anatomical structures such as the ribs, lungs, and heart exacerbates the complexity. To solve this predicament, we have devised a systematic end-to-end pipeline harnessing deep learning using our SCOLIONET’s customized CNN architecture. Our innovative approach aims to automate the CA identification, thus, addressing the inconsistencies of traditional methods. We capitalized on the capabilities of AI in assisting human perception.

Our overall findings have been profound, showing notable consistency between experts and machine learning estimation, with an average difference of 2.86 degrees, a remarkably reliable value that significantly reduces the standard manual variations. In essence, our established framework has made a vital contribution to machine-driven medical imaging examination. The application of this research directly impacts the clinical sphere for rapid, accurate, and reliable means of scoliosis severity evaluations. Consequently, our discoveries are a stride in offering a simplified and robust approach to empower medical personnel with the modern tools needed to understand scoliosis and enhance patient care comprehensively.

As for future work, we are committed to refining our models’ precision through numerous strategies, such as enhancing the segmentation algorithm and evaluating the performance of other CNN networks. The authors also plan to implement federated learning approaches, where models are trained locally on distributed devices, as this will allow for collaborative model training without sharing of sensitive (private) patient data and to explore collaboration with medical research institutions to obtain more datasets in accordance with data sharing ethical practices.

## Figures and Tables

**Table 1:** CA measurements per severity (source: Cobb method, adapted from ref. [4]).

No.	Angle in Degrees	Spine Class
1	0–10	Normal
2	10–20	Mild
3	20–40	Moderate
4	>40	Severe

**Table 2:** Average quality enhancement of selected images (N = 100).

Method	Correlation Coefficient (CC)	Spearman Rank Order Correlation Coefficient (SROCC)
VIF	0.973	0.968

**Table 3:** Computing performance of different deep neural networks with reference to the number of processors.

Deep Neural Network Model	Training Time in Minutes	
	Four Active Processor Cores	Eight Active Processor Cores
Standard U-Net	21.16	18.35
Residual U-Net	23.81	20.15
SCOLIONET	24.48	19.38

**Table 4:** Computing performance of different deep neural networks with reference to memory consumptions.

Deep Neural Network Model	Memory Consumption Based on Number of Batches						
	1	2	4	8	16	32	64
Standard U-Net	0.67	0.63	0.67	0.67	0.67	0.66	0.68
Residual U-Net	0.72	0.66	0.72	0.72	0.71	0.73	0.78
SCOLIONET	0.72	0.65	0.71	0.70	0.70	0.72	0.77

Note: eight active processor cores.

**Table 5:** Cross-validated segmentation performance of the three neural network architectures based on various metrics.

Fold	SDC			IoU			MSE		
	U-Network	SCOLIONET	RU-Network	U-Network	SCOLIONET	RU-Network	U-Network	SCOLIONET	RU-Network
1	0.939 ± 0.034	0.978 ± 0.027	0.952 ± 0.025	0.923 ± 0.042	0.967 ± 0.028	0.940 ± 0.045	0.031 ± 0.018	0.021 ± 0.014	0.028 ± 0.018
2	0.943 ± 0.035	0.973 ± 0.025	0.953 ± 0.024	0.921 ± 0.045	0.963 ± 0.027	0.942 ± 0.040	0.032 ± 0.015	0.025 ± 0.015	0.029 ± 0.016
3	0.941 ± 0.031	0.975 ± 0.026	0.951 ± 0.029	0.922 ± 0.044	0.960 ± 0.028	0.939 ± 0.039	0.033 ± 0.016	0.024 ± 0.016	0.030 ± 0.018
4	0.942 ± 0.034	0.976 ± 0.027	0.949 ± 0.026	0.923 ± 0.045	0.969 ± 0.031	0.941 ± 0.041	0.034 ± 0.017	0.025 ± 0.014	0.032 ± 0.017
5	0.942 ± 0.035	0.977 ± 0.028	0.951 ± 0.027	0.921 ± 0.041	0.961 ± 0.029	0.942 ± 0.043	0.032 ± 0.015	0.027 ± 0.015	0.033 ± 0.019
6	0.943 ± 0.033	0.975 ± 0.024	0.949 ± 0.028	0.929 ± 0.046	0.964 ± 0.030	0.940 ± 0.043	0.033 ± 0.019	0.029 ± 0.020	0.030 ± 0.016
7	0.941 ± 0.032	0.976 ± 0.027	0.950 ± 0.029	0.925 ± 0.043	0.962 ± 0.030	0.948 ± 0.040	0.032 ± 0.017	0.028 ± 0.016	0.032 ± 0.018
8	0.942 ± 0.032	0.977 ± 0.028	0.951 ± 0.030	0.931 ± 0.045	0.965 ± 0.032	0.946 ± 0.039	0.034 ± 0.015	0.027 ± 0.013	0.031 ± 0.016
9	0.941 ± 0.035	0.978 ± 0.026	0.950 ± 0.028	0.932 ± 0.048	0.968 ± 0.033	0.945 ± 0.040	0.033 ± 0.016	0.023 ± 0.012	0.031 ± 0.018
10	0.940 ± 0.037	0.974 ± 0.025	0.949 ± 0.029	0.933 ± 0.049	0.964 ± 0.031	0.943 ± 0.041	0.032 ± 0.018	0.024 ± 0.017	0.029 ± 0.016
Mean ± Standard deviation	0.941 ± 0.033	0.975 ± 0.026	0.950 ± 0.027	0.926 ± 0.044	0.963 ± 0.029	0.942 ± 0.041	0.032 ± 0.016	0.025 ± 0.015	0.030 ± 0.017
Training duration, eight processing cores (in minutes):				SCOLIONET (19.38), RU-Network (20.15), and U-Network (18.35)					
Test duration, eight processing cores (in seconds):				SCOLIONET (0.04), RU-Network (0.03), and U-Network (0.02)					

**Table 6:** Comparative calculation of Cobb angles among healthcare professionals (manual) vs. SCOLIONET (automated).

X-ray ID	SCOLIONET’s Cobb Angle			Experts’s Cobb Angle (Observed)			Absolute Difference of Vertebral References (SCOLIONET vs. Expert)		
	Most Tilted Upper Vertebrae	Most Tilted Lower Vertebrae	Cobb Angle Degree	Most Tilted Upper Vertebrae	Most Tilted Lower Vertebrae	Cobb Angle Degree	Most Tilted Upper Vertebrae	Most Tilted Lower Vertebrae	Cobb Angle Degree
0021	TH08	LU03	23.80	TH08	LU03	23.50	TH08—0	LU03—0	0.30
0055	TH12	LU02	12.50	TH12	LU02	13.70	TH12—0	LU02—0	1.20
0071	TH09	LU04	13.60	TH09	LU03	15.20	TH09—0	LU04/LU03—1	1.60
0085	TH05	TH11	25.30	TH05	TH11	26.20	TH05—0	TH11—0	0.90
0103	TH06	TH12	24.60	TH06	TH12	24.50	TH06—0	TH12—0	0.10
0123	TH10	LU03	23.10	TH10	LU03	22.90	TH10—0	LU03—0	0.20
0203	TH03	TH09	41.20	TH02	LU01	43.70	TH03/TH07—3	TH09/LU01—3	3.50
0233	TH05	LU04	32.60	TH05	LU04	32.50	TH05—0	LU04—0	0.10
0253	TH05	LU02	40.60	TH06	LU04	42.50	TH03/TH06—2	LU02/LU04—2	3.00
0313	TH06	TH12	16.70	TH06	TH12	17.20	TH06—0	TH12—0	0.50
Legend:						TH01–TH12 (thoracic), LU01–LU05 (lumbar) [1]			
T-test (SCOLIONET vs. Experts)						t = 0.1713, *p*-value = 0.8659 (Not significant at *p* < 0.05)			
MAPE (SCOLIONET vs. Experts)						3.86% (Accuracy = 96.13)			
Mean absolute difference of measurements (SCOLIONET vs. Experts)						2.86 degrees			

**Table 7:** Comparative vertebra segmentation accuracies using various methodologies.

Architectures/Approaches/Models/Mechanisms	Accuracy
Standard U-Net (with different configurations) [22]	88.01%
Patch-wise portioning + minimum bounding boxes [14]	88.60%
K-Means clustering + regression [15]	88.20%
Residual U-Net [23]	88.30%
Lateral boundary detection [16]	88.50%
3-Stage process (Otsu algorithm, morphology, and polynomial fitting) [11]	90.20%
Otsu thresholds + Hough transformations [17]	90.30%
Corner tracking + support vector machines [18]	90.40%
Dense U-Net [23]	94.20%
Residual U-Net (polynomial fitting + minimum border box) [24]	95.10%
SCOLIONET (spinal isolation via Adboost + color shifting + SWF + polynomial fitting + bounding box method + modified U-Net with atrous spatial pyramid pooling)	97.50%

## Data Availability

The data presented in this study are publicly available: (a) SpineWeb (collaborative platform for research on spine imaging and image analysis: dataset 16: 609 spinal anterior–posterior X-ray images); (b) a dataset of scoliosis, spondylolisthesis, and normal vertebra X-ray images (DOI: 10.17632/xkt857dsxk.1).
